# A Distinctive microRNA (miRNA) Signature in the Blood of Colorectal Cancer (CRC) Patients at Surgery

**DOI:** 10.3390/cancers12092410

**Published:** 2020-08-25

**Authors:** Jessica Gasparello, Chiara Papi, Matteo Allegretti, Elena Giordani, Fabio Carboni, Settimio Zazza, Edoardo Pescarmona, Paolo Romania, Patrizio Giacomini, Chiara Scapoli, Roberto Gambari, Alessia Finotti

**Affiliations:** 1 Department of Life Sciences and Biotechnology, Ferrara University, 44121 Ferrara, Italy; gspjsc@unife.it (J.G.); chiara.papi@unife.it (C.P.); chiara.scapoli@unife.it (C.S.); 2 Oncogenomics and Epigenetics, IRCCS (Istituto di Ricovero e Cura a Carattere Scientifico) Regina Elena National Cancer Institute, 00144 Rome, Italy; matteo.allegretti@ifo.gov.it (M.A.); elena.giordani@ifo.gov.it (E.G.); pabepabe@gmail.com (P.R.); patrizio.giacomini@ifo.gov.it (P.G.); 3 Digestive Surgery, IRCCS (Istituto di Ricovero e Cura a Carattere Scientifico) Regina Elena National Cancer Institute, 00144 Rome, Italy; fabio.carboni@ifo.gov.it (F.C.); settimio.zazza@ifo.gov.it (S.Z.); 4 Pathology, IRCCS (Istituto di Ricovero e Cura a Carattere Scientifico) Regina Elena National Cancer Institute, 00144 Rome, Italy; edoardo.pescarmona@ifo.gov.it

**Keywords:** circulating miRNA, liquid biopsy, colorectal cancer, next generation sequencing, droplet digital PCR

## Abstract

Background: Liquid biopsy (LB) provides an examination of the peripheral blood of cancer patients for circulating tumor cells, cell-free nucleic acids and microRNAs (miRNAs) and is an established tool of precision medicine. Unlike most previous LB studies that focused on advanced metastatic colorectal cancer (CRC), we assessed miRNA dysregulation in blood samples obtained on the day of surgery from patients with primary CRC lesions but no clinical evidence of extra-colonic diffusion. In this study, plasma preparation included miRNAs associated to exosomes, but excluded large macrovesicles from the preparation. Methods: The miRNA profile in plasma isolated from a cohort of 35 CRC patients at the day of surgery was analyzed by Next Generation Sequencing (NGS) and further confirmed by droplet digital RT-PCR (dd-RT-PCR). Results: A miR-141-3p/miR-221-3p/miR-222-3p upregulation signature previously described in advanced CRC did not discriminate the analyzed early-CRC cohort from six tumor-free donors (Tf-D). In contrast, NGS-based miRNome analysis of a training cohort of five CRC and three tumor-free donors identified a novel, distinct nine miRNA signature comprising five up-regulated and four down-regulated miRNAs, six of which could be confirmed in the full CRC and tumor-free donor validation dataset by dd-RT-PCR. Additionally, a *KRAS* (Kirsten Rat Sarcoma Viral Oncogene Homolog) mutant status was correlated with the plasma content of three identified miRNAs. Conclusions: When the data obtained were comparatively evaluated, at least one of the miRNAs belonging to the signature list was found to be dysregulated in 34/35 (97.1%) of our early-CRC plasma samples. The miRNA list provides diagnostic markers as well as possible molecular targets for protocols focusing on “microRNA therapeutics”.

## 1. Introduction

Colorectal cancer (CRC) is the third most common malignancy and the fourth leading cause of cancer death worldwide [1,2,3,4]. Its burden is expected to increase by 60%, with more than 2.2 million new cases and 1.1 million cancer deaths by 2030 [5,6]. Prevention and early diagnosis are key to counter this trend. Stool and blood tests to identify methylated DNA are among the preferred methods for the screening of pre-symptomatic and pauci-symptomatic CRC patients [7]. However, they are far from optimal, opening several issues, such as proband compliance, costs, standardization, and false negatives/positives. These limitations are widely recognized, particularly by patient advocacy organizations (https://digestivecancers.eu). Thus, early-stage CRC detection still remains a major unmet need in cancer diagnostics.

Liquid Biopsy (LB)—i.e., the identification of molecular cancer alterations in biological fluids, is expected to significantly improve test compliance and specificity, as compared to stool examinations [8,9,10,11,12,13,14,15]. For instance, CancerSEEK, a blood-based LB assay to assess the tumor-bearing status of patients at surgery, achieves a remarkable sensitivity (>80% in the case of CRC) by implementing circulating tumor DNA (ctDNA) and blood protein biomarkers [16]. Unsurprisingly, many groups have applied similar approaches to discover other cancer-associated analytes, circulating microRNAs (miRNAs) being a preferred study subject. Nevertheless, the majority of these groups have been focused on advanced, metastatic CRC [17,18,19], with few exceptions [20]. MicroRNAs are short non-coding RNA molecules acting as gene regulators. They either repress translation or induce the cleavage of target RNA transcripts [21,22,23]. Emerging evidence suggests that the altered expression of miRNA may be involved in the pathogenesis of cancer [24,25]. In particular, oncomiRNAs and metastamiRNAs can be up-regulated in cancer and this mediates the down-regulation of target onco-suppressor RNAs [26]. Conversely, tumor-suppressor miRNA can be down-regulated in cancer and this feature is associated with the up-regulation of tumor-causing genes. Therefore, miRNAs are candidates as tumor-associated markers in several diagnostic procedures (including LB) and may represent appealing therapeutic targets. Inhibitions of up-regulated oncomiRNAs and mimicking the biological activity of down-regulated tumor-suppressor miRNAs have both been described [27,28,29].

Despite considerable success, most approaches focus on miRNA dysregulated in advanced, metastatic CRC [17,18,19], with few exceptions [20]. This is unfortunate, because capturing earlier dysregulation may considerably expand miRNA application. For this reason, we decided to focus on non-metastatic patients at earlier disease stages—e.g., on a population of CRC patients very similar to the CancerSEEK population. These patients had recently received a CRC diagnoses and were screened by total-body medical imaging to exclude cancer dissemination. Thus, the primary tumor is bona fide the only detectable cancer, which makes them eligible for surgical removal. Taking blood on the day of surgery offers the possibility to explore CRC in an immediate post-diagnostic setting.

Herein, we investigated the blood accumulation of three miRNAs (miR-141-3p, miR-221-3p and miR-222-3p) previously shown to be elevated in late CRC stages [30,31,32]. Next, we investigated the circulating miRNome at surgery, looking for alternative CRC-associated miRNAs, and ultimately short-listed nine up-/down-regulated miRNAs. They make up a novel molecular signature that does not overlap with previous miRNA signatures, outperforms the miR-141-3p/miR-221-3p/miR-222-3p combination (even when this comparison is restricted to three up-regulated miRNAs extracted from the 9-miRNA signature), and comprises new candidates for CRC diagnosis and targeted therapy. In this study, plasma preparation included miRNAs associated with exosomes but excluded large macrovesicles from the preparation. Finally, we investigated whether a correlation does exist between CRC-associated miRNAs and Kirsten Rat Sarcoma Viral Oncogene Homolog (*KRAS*) status. *KRAS* status was selected since, in the Consensus Molecular Subtype classification [33], CRC tumors are stratified in four groups and *KRAS* mutations are over-represented in the aggressive-upon-relapse CMS3 subtype. Moreover, high *KRAS* ctDNA levels are predictive of a poor CRC outcome [34].

## 2. Results

### 2.1. Plasma Content of miR-141, miR-221 and miR-222 in CRC Patients

**Figure 1:**
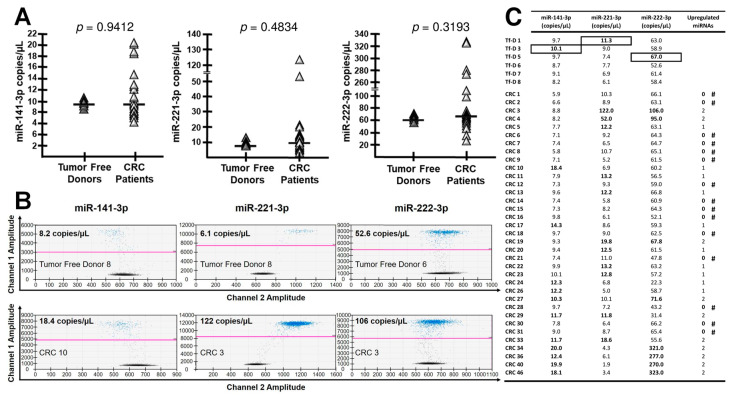
Digital Droplet RT-PCR (dd-RT-PCR) of miR-141, miR-221 and miR-222 in CRC patients. (**A**): Plasma samples from 35 CRC patients (CRC) and 6 tumor-free donors (Tf-D) were assessed by dd-RT-PCR. Representative plots are depicted in panel (**B**) and a summary in panel (**C**). (**C**): the highest values in Tf-D control samples are black boxed, and denote up-regulation in the CRC samples compared to the highest control. The number of up-regulated miRNAs in CRC patients (indicated in bold) is noted. # = no miRNA up-regulation.

MicroRNAs miR-141-3p, miR-221-3p and miR-222-3p were shown to be elevated in the blood of CRC patients [31,32]. However, this evidence comes from three independent studies carried out on patients at advanced disease stages [35,36,37]. On this basis, we set out to determine, in a single study, whether these three miRNAs (alone and in aggregate) might be similarly elevated at earlier disease stages—i.e., in the presence of a more limited tumor burden. To this end, we enrolled 35 newly diagnosed CRC patients. Blood was obtained right before (hours to minutes) surgical removal of the primary tumor, and plasma miRNAs were quantitatively assessed by dd-RT-PCR assays specifically designed for each miRNA. Blood from six tumor-free donors (Tf-D) was assessed in parallel. The results are shown in graphical form (Figure 1A), as representative dd-RT-PCR plots (Figure 1B), and in tabular form (Figure 1C). The plasma levels of miR-141-3p, miR-221-3p and miR-222-3p exceeded the highest levels seen in the controls (boxed values) in only 11/35 (31.4%), 11/35 (31.4%) and 8/35 (22.8%) of the tested patients, respectively (bolded in Figure 1C). Even when cumulatively considered, the three miRNAs were poorly informative on our samples, since only 20/35 (57.1%) CRC patients displayed at least one up-regulated miRNA, whereas the remaining patients displayed no up-regulation at all (Figure 1C). We conclude that neither single miRNAs nor their combination efficiently discriminate CRC patients from tumor-free donors (Tf-D) suggesting that miR-141-3p, miR-221-3p and miR-222-3p may find limited application at early CRC stages, unless combined with the analysis of other miRNAs. In view of this, we were prompted to undergo a systematic miRNome screening to look for more robust miRNA dys-regulation events in the pre-operative stage.

### 2.2. miRNome Assessment by NGS: Experimental Training Set

Five and three plasma samples were randomly selected from CRC patients and tumor-free donors (Tf-D), respectively. These eight samples were used for NGS-based miRNome assessment as described in the Methods. Data were analyzed to highlight clusters, if any, with possible biological significance.

**Figure 2:**
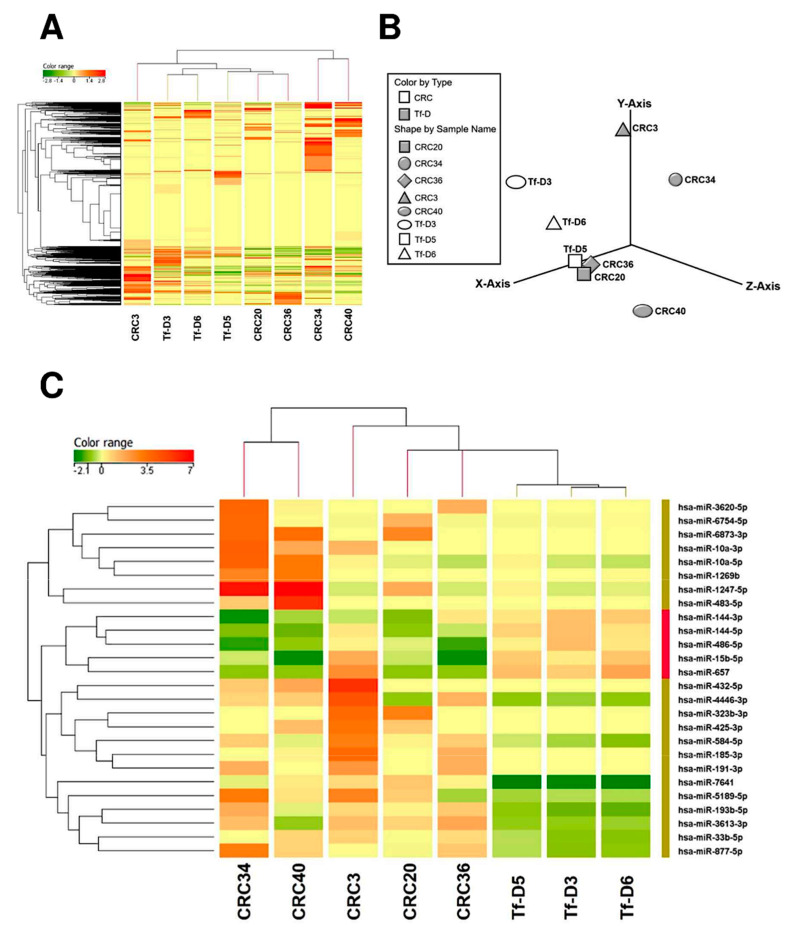
miRNome analysis. (**A**): Hierarchical clustering of the miRNAs identified in each expression of library pattern. The miRNAs were from tumor-free donors (Tf-D) and colorectal cancer (CRC) patients’ blood samples. Green = down-regulation; red = up-regulation. (**B**): Profile of overall miRNAs expression changes. The differential expression levels of miRNA in library (CRC) versus healthy groups. Each shape in the 3D visualization represents a group of miRNAs. Principal component analysis (PCA) captured the variation observed in the experiment in the first three principal components (PC). (**C**): The heatmaps of CRC samples versus tumor-free plasmas are shown in Figure S1 (FC > 1.5) and in Figure 2C (FC > 2).

**Figure 3:**
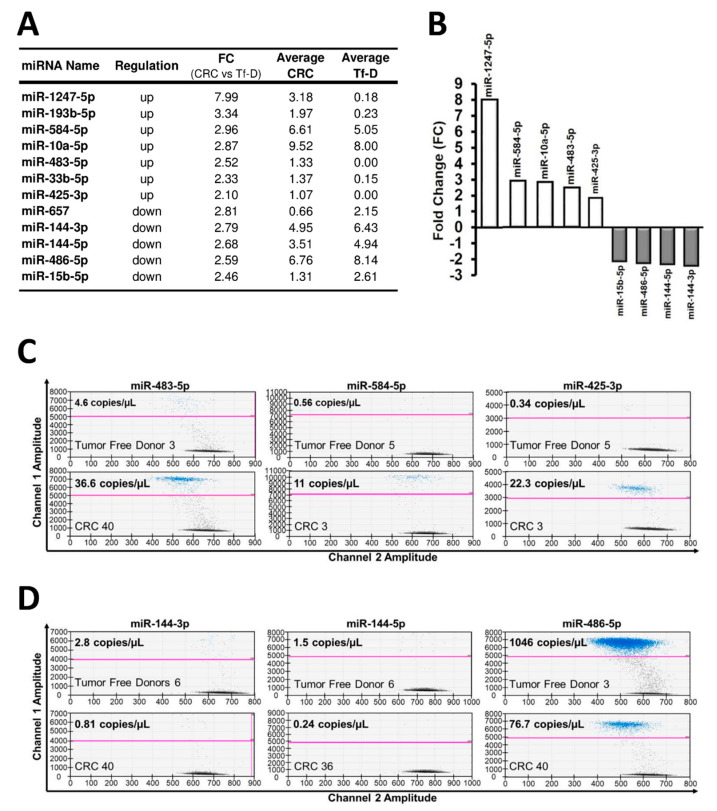
miRNA selected for digital droplet RT-PCR validation. (**A**,**B**): List of the candidate miRNAs chosen for dd-RT-PCR validation. Up- and down-regulation levels are indicated. (**C**,**D**): Representative plots of dd-RT-PCR analysis. The full dd-RT-PCR data (copies/µL) are reported in Figure 4.

The unsupervised heatmap of log transformed normalized miRNA expression levels, the unsupervised Principal Component Analysis (PCA), as well as the heatmaps of CRC vs. tumor-free donors (at Fold Changes >2) are shown in Figure 2—panels A, B and C, respectively. The heatmap of CRC vs. tumor-free samples at Fold Changes >1.5 is shown in Figure S1. All these elaborations provided evidence for distinct clusters of CRC vs. tumor-free donors (Tf-D), although clusters were not entirely resolved. As to individual miRNAs, setting the FC cutoff at >1.5 resulted in the identification of 77 differentially expressed miRNAs (Figure S1), a number that was refined to 26 by repeating the analysis at FC value >2.0 (Figure 2C). Next, we excluded miRNAs that (a) exhibited the greatest variation (ranges) across Tf-D samples (SD >2.5 fold the average values), and/or (b) were expressed at very low levels in dd-RT-PCR (content <0.2 copies/µL) or for which an amplification kit was not available. This resulted in a short list of candidate miRNAs in our training set (Figure 3A) comprising the final 9-miRNA signature, including five up-regulated and four down-regulated miRNAs (Figure 3B). MicroRNA-specific dd-RT-PCR assays were developed to independently confirm this signature in a larger cohort of CRC samples. Representative plots of dd-RT-PCR analysis of up- (Figure 3C) and down- (Figure 3D) regulated miRNAs are shown in Figure 3.

### 2.3. dd-RT-PCR Assessment of the 9-miRNA CRC Signature on the Full Experimental Dataset

**Figure 4:**
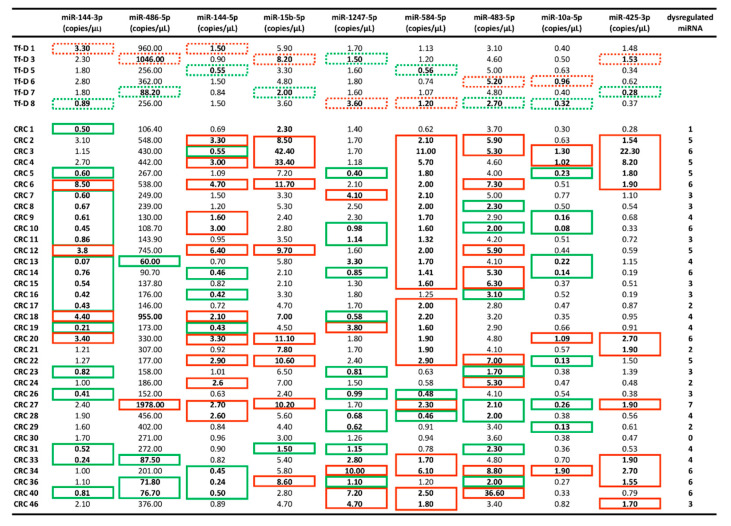
miRNA content in plasma samples. Plasma miRNAs levels (copies/µL) in Tumor-free Donors (from Tf-D1 to Tf-D8) and CRC (from CRC1 to CRC46) plasma samples. Values detected by dd-RT-PCR. The dotted green and red boxes in the control samples indicate the plasma lowest (green) and highest (red) miRNA levels used for comparison with the CRC samples. In the CRC samples, the solid green boxes show down-regulated miRNA values (lower values with respect to the lowest values found in controls), while the solid red boxes show up-regulated miRNA values (higher values with respect to the highest values found in controls). The number of dysregulated miRNAs is indicated from each CRC sample in the last right columns (this numbers in several CRC samples increase considering the analysis of miR-141-3p, miR-221-3p and miR-222-3p shown in Figure 1C). A further analysis comparing CRC values with the average values found in controls is reported in Figure S2. A comparison of miR-15b-5p, miR-584-5p and miR-425-3p (displaying an upregulation trend in CRC samples), and miR-141-3p, miR-221-3p and miR-222-3p, is shown in Table S3.

**Figure 5:**
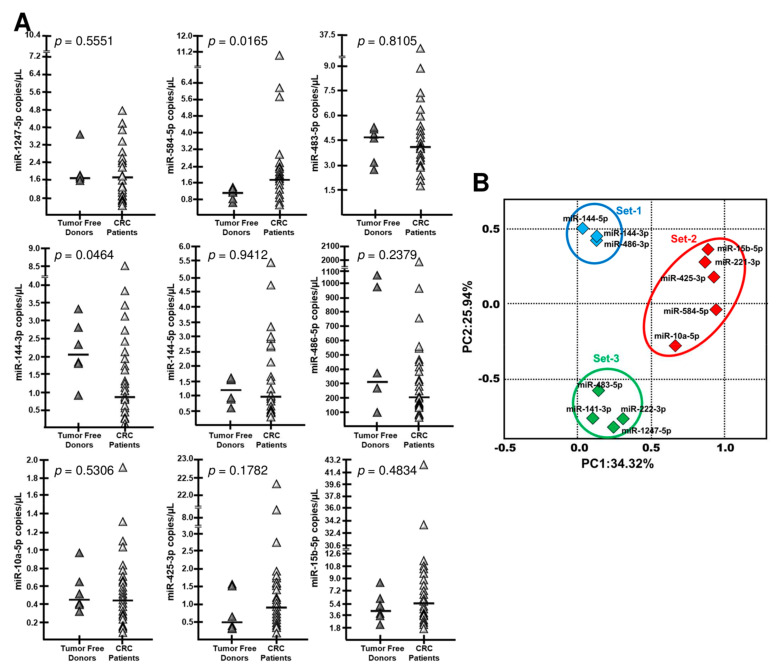
Digital droplet RT-PCR validation and principal component analysis (PCA). (**A**): Digital droplet RT-PCR validation (dd-RT-PCR) comparing plasma samples from 35 CRC patients and 6 tumor-free donors with respect to the content of dysregulated miRNAs. (**B**): PCA captured the variation observed in the experiment in the first three principal components (PC). Three principal microRNA sets on the basis of their respective correlations can be proposed, as depicted in the panel. The nine miRNAs of the proposed signature, plus the miR-141-3p, miR-221-3p and miR-222-3p, were considered.

Figure 4 and Figure 5A summarize the complete quantitative analysis of the 9-miRNA signature in the plasma of tumor-free donors (Tf-D) and CRC samples (*n* = 6 and *n* = 35, respectively). When compared to the training set, the full dataset revealed a wider scatter in the observed values, as expected. Nevertheless, overall trends (increases in miR-584-5p, miR-15b-5p, and miR-425-3p and decreases in miR-144-3p, miR-144-5p, and miR-486-5p in CRC compared to normal) could be confirmed in at least 6/9 miRNAs. The two most significant changes in miRNA levels were seen for miR-584-5p (up-regulated) and miR-144-3p (down-regulated), Mann–Whitney *p* = 0.016 and *p* = 0.046, respectively. Data are analytically displayed in Figure 4 as copies/uL of miRNA in plasma, and the dysregulation (up- or down-regulation relative to control) is individually noted for each patient. Although single miRNAs in the 9-miRNA CRC signature may not be informative in individual patients (the most informative was miR-584-5p, that was found upregulated in 25/35 patients), it is readily evident from Figure 4 that, in all but one (CRC30) of the 35 tested CRC samples, there are from 2 to 8 dysregulated miRNAs. Therefore, the 9-miRNA signature is informative as to the CRC disease status at surgery in 97.1% of the CRC patients tested in this study. The data depicted in Figure 4 were supported by a further analysis conducted comparing the values in CRC samples with the average values found in controls and reported in Figure S2. Of interest, some individual CRC plasma samples behaved as outliers either because of the number of dysregulated miRNAs or because of very high down- or up-regulation. In summary, there are some CRC cases with strong signatures. This can be a very useful information in the road of personalized treatments based of the so called “miRNA therapeutics”.

Principal component analysis (PCA) on the data shown in Figure 1 and Figure 5A captured the 60.3% of variation observed in the reported experiments. PCA grouped the nine miRNAs in the signature in three distinct, widely separated sets (Figure 5B). Three principal miRNA sets are detectable on the basis of their respective positions—i.e., miR-144-5p, miR-144-3p, miR-486-5p (Set-1), miR-15b-5p, miR-221-3p, miR-425-3p, miR-584-5p, miR-10a-5p (Set-2) and miR-483-5p, miR-141-3p, miR-222-3p, miR-1247-5p (Set-3) (Figure 5B). There were significant and strong correlations among miRNAs within set 1 (Sperman’s R *p*-values <0.004) as well as Set 2 (Sperman’s R *p*-values ranging from 0.001 to 0.03). In summary, these data suggest the co-regulation of the nine selected miRNAs in three distinct clusters. In order to identify a signature able to promptly detect patients at early CRC stages, we selected, from each set obtained through PCA, the most informative miRNA, namely: miR-144-3p (Set-1), miR-584-5p (Set-2)—both significantly dysregulated—and miR-1247-5p (Set-3), showing a FC value of around 8.0 in the dd-RT-PCR validation analysis. From Figure 4, 31 of the 35 tested CRC patients (88.6%) showed at least two of these three miRNAs dysregulated (last column on the right), whereas miRNAs miR-141, miR-221 and miR-222 showed at least two miRNAs dysregulated in only 23/35 (65.7%) patients (Figure 4, last right column), a proportion significantly lower than the former (Z-score 2.277, *p*-value 0.023).

In addition, we performed a comparative analysis of miR-15b-5p, miR-584-5p and miR-425-3p (displaying an upregulation trend in CRC samples, as clearly evident from Figure 4) and miR-141-3p, miR-221-3p and miR-222-3p (Table S1), demonstrating that at least one of the three miRNAs extracted from the 9-miR signature is up-regulated in 28/35 (80.00%) CRC samples; by contrast, in the case of miR-141-3p, miR-221-3p and miR-222-3p, at least one miRNA was found up-regulated in 25/35 (71.43%) CRC samples. Interestingly, when these six miRNAs are considered together, at least one miRNA was found up-regulated in 33/35 (94.28%) CRC samples. Therefore, the diagnostic power of miR-141-3p, miR-221-3p and miR-222-3p might be improved by using all or part of our 9-miR signature (Table S1).

Concerning the high levels of CRC heterogeneity with respect to miRNA content (Figure 4 and Table S2), further analyses are necessary, since the analyses of tumor location, grading and the presence of a metastatic phenotype create a need for larger numbers of recruited patients.

### 2.4. Association between miRNA Plasma Levels and Kirsten Rat Sarcoma Viral Oncogene Homolog (KRAS) Mutations

**Figure 6:**
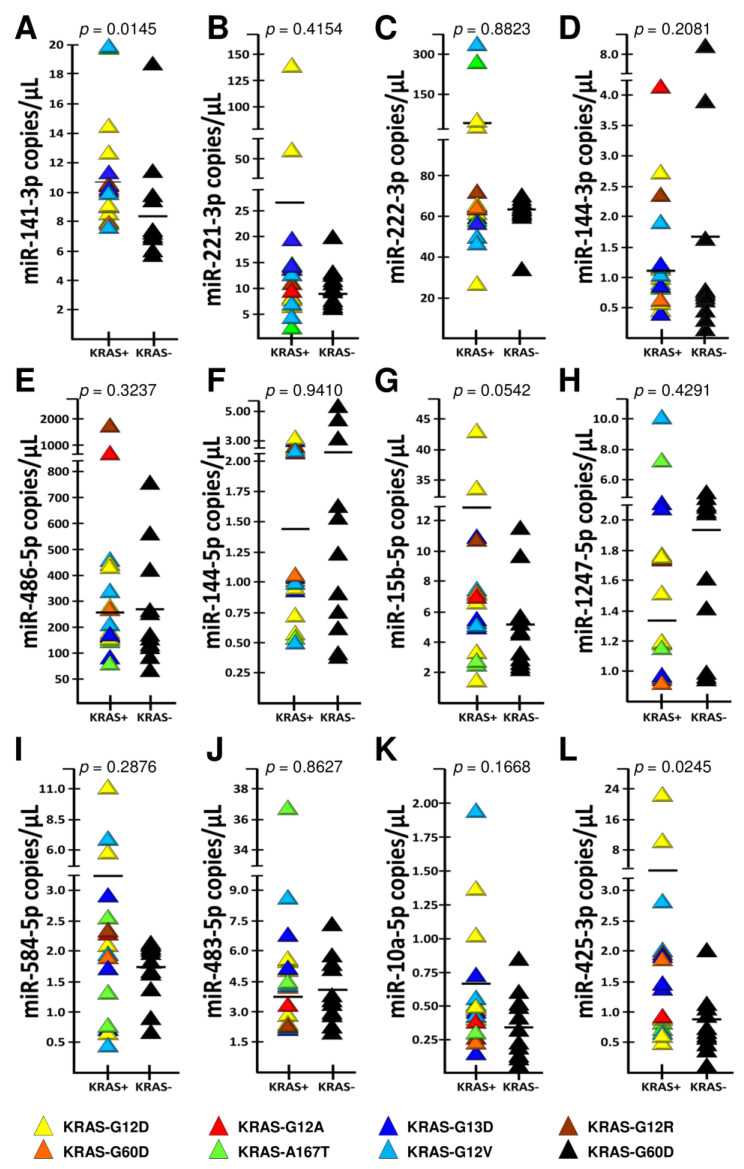
Correlation between miRNA dysregulation and *KRAS* mutations. (**A**–**L**): dd-RT-PCR comparison of plasma samples from *KRAS*-MUT vs. *KRAS*-WT CRC patients (see Figure 4) with respect to content of dys-regulated miRNAs, including miR-141-3p (**A**), miR-221-3p (**B**) and miR-222-3p (**C**).

The possible association between the miRNA plasma levels depicted in Figure 6 and a *KRAS* mutant status was determined in consideration of the more aggressive phenotype of *KRAS*-mutated CRC cancers [38,39,40,41]. To this end, we divided our patient population in two groups: the former bearing *KRAS*-WT tumors (*n* = 11), and the latter carrying the p.G12D (*n* = 5), p.G12V (*n* = 3), p.G13D (*n* = 3), p.G12A (*n* = 2), p.G60D (*n* = 1), p.A146T (*n* = 1) and p.G12R (*n* = 1) *KRAS* mutations (see Table S2). Figure 6 shows the miRNA plasma levels in the two CRC groups, and shows that miRNAs miR-425-3p and miR-141-3p were significantly higher in the 16 *KRAS*-mutated patients, as compared to the 11 patients in the WT group (Mann–Whitney *p* = 0.025 e *p* = 0.015, respectively). For miR-15b-5p, a borderline *p*-value was observed (Mann–Whitney *p* = 0.054). Thus, it is concluded that miRNA dysregulation (miR-425-3p and miR-141-3p, and possibly miR-15b-5p) is associated with *KRAS* mutation. The non-significant *KRAS*-association trend of miR-221-3p, miR-486-5p, miR-1247-5p and miR-584-5p needs confirmatory future studies on higher numbers of CRC patients.

## 3. Discussion

In this study, we show that three blood miRNAs (miR-141, miR-221 and miR-222), long known to be upregulated in advanced CRC, are weakly associated with early CRC. These three miRNAs were found to be upregulated in 65.7% of CRC patients at surgery (Figure 1C), whereas the remaining miRNA levels are very similar (or lower) in samples from tumor-free donors (Tf-D). Based on these results, we went on and identified a novel 9-miRNA signature from the blood miRNomes of CRC patients with localized disease, on the day of surgical removal of their primary tumor, days to weeks after the initial diagnosis. This early-CRC signature, identified by NGS, was subsequently validated by dd-RT-PCR, and displayed 97.1% specificity (Figure 4 and Figure S2. The 9-miRNA is independent of the above mentioned miR-141-3p, miR-221-3p and miR-222-3p but can complement the diagnostic power of these three miRNAs (Table S1). Altogether, these results highlight the importance of implementing stage-specific CRC biomarkers. Improved association with a disease status held, even when the analysis was restricted to the upregulated miRNAs of the newly identified signature.

There are several caveats to these findings, including the limited number of clinical samples: tumor-free donors (Tf-D) and CRC patients were 3 and 5, and 6 and 35, in the training and experimental datasets, respectively. Therefore, it is not expected that this signature might be self-standing, but rather that it represents a useful source of biomarkers to be added to other classifiers and predictors. In addition, further studies will clarify the role (if any) of several miRNAs that have been excluded by our analysis (for instance due to their low expression in the training set or due to the lack of commercial PCR kits). Notwithstanding these limitations, we provide evidence that the 6-miRNA signature is robust, is consistent with a large body of published evidence, and captures early dysregulation events in CRC. As assessed by PCA, dysregulation appears to involve a minimum of three distinct miRNA sets of related biological functions mapped by miR-144-5p, miR-144-3p and miR-486-5p (Set 1), miR-15b-5p, miR-221-3p, miR-425-3p, miR-584-5p and miR-10a-5p (Set 2), miR-483-5p, miR-141-3p, miR-222-3p and miR-1247-5p (Set 3). Of special interest, although only one of these miRNAs was previously associated in blood with CRC, and none with an early-stage disease, many were suspected to be strongly involved in CRC pathobiology [42,43,44,45]. For instance, miR-144-3p (down-regulated in 25/35 of our CRC patients) was also expressed at low levels in the SW837 and SW1463 cell lines, and its overexpression suppresses CRC cell viability, migration and proliferation [46]. It is tempting to speculate that low miR-144-3p blood levels are due to the adaptive selection of aggressive CRC cell populations. Potential targets of miR-144-3p in CRC are anoctamin 1 (ANO1) [47], MAD Homolog 4 (SMAD4) [48] and Rho-associated coiled-coil containing protein kinase 1 (ROCK1) [46,49]. Likewise, miR-486-5p down-regulation in blood mirrors its low expression (relative to normal), previously seen in tumor tissues from early as well as advanced CRC stages [50]. Pleomorphic adenoma gene like-2 (PLAGL2) [51], a zinc finger protein transcription factor displaying oncogenic function in colorectal cancer (CRC) has been proposed as a target of miR-486-5p. As to the down-regulation of miR-1247 in 11/35 of our CRC patients, it is similarly consistent with a predominant tumor suppressor function that is well established in a variety of tumors, including CRC [52,53,54,55,56,57]. The MYCBP2/c-myc axis may underlie the anti-tumor activities of miR-1247 [58]. However, we also observed up-regulation in seven cases, a finding that deserves further functional studies.

The upregulation of miRNAs in our blood collection is also in keeping with previous studies. For instance, miR-483-5p has been shown to be strongly oncogenic by acting through a complex interplay involving Insulin Growth Factor (*IGF2*) and Deleted in Liver Cancer (*DLC-1*) [52]. Interestingly, this miRNA was already known to be higher in the blood of CRC patients as compared to normal controls [52]. As to miR-10a-5p and miR-425-3p, little is known about their biological role, but they are highly expressed in tumors of the right colon [59], and *BRAF*-mutated CRCs [60], respectively. Thus, with the exception of miR-144-5p, miR-584-5p, and miR-15b-5p, that, to our knowledge, were not previously associated with CRC, all the miRNAs identified by the present study are contained in the known CRC tissue miRNome, and behave as established CRC onco-suppressors (when down-regulated) or oncomiRNAs (when up-regulated). Concordance between the presently detected levels in blood and the previously noted levels in CRC tissues suggests, albeit in the absence of direct testing, a simple straightforward mechanisms whereby direct release from CRC tumors results in roughly proportional miRNA abundance in CRC tissues and blood. This is remarkable, since several confounding factors appear to operate on the fine tuning of miRNA and ctDNA release, as assessed by mouse CRC xenotransplant models [61]. Possible molecular targets of the miRNAs belonging to the CRC-9-miRNA-signature are shown in Table S3.

In analogy with many published studies [62,63,64], some of our up-regulated miRNAs may be useful therapeutic targets. In this respect, it is important to note that *KRAS* mutations are predictive of resistance to epidermal growth factor receptor (EGFR)-blockade with therapeutic antibodies in metastatic CRC. Furthermore, *KRAS* mutations are over-represented in the aggressive-upon-relapse CMS3 subtype. Moreover, high *KRAS* ctDNA levels are predictive of a poor CRC outcome [34]. Interestingly, we found that miR-425-3p and miR-141-3p, and possibly miR-15b-5p, are over-represented in the *KRAS*-mutated "undruggable" subset. Fortunately, several new agents are now being developed for this subset, and it will be of interest to determine how miRNA dys-regulation intertwines with *KRAS*-dependent constitutive activation of the *EGFR* pathway [65]. Our findings prioritize future experiments of combined KRAS/miRNA targeting in a KRAS-mutated background.

Concerning the possible association of the 9-miR signature with other mutations, our cohort of CRC patients are not informative for NRAS and BRAF mutations (Table S2). A larger and informative cohort of patients will be necessary. In addition, the miRNA association with other markers (such as Ki-67 and CD34) should be considered, in order to determine the impact of miRNA expression on cell proliferation in early CRC stages.

## 4. Materials and Methods

### 4.1. Study Design and Ethics

This study enrolled 35 patients diagnosed as CRC patients at IRCCS Regina Elena National Cancer Institute, Rome, Italy. Demographic and clinical features are shown in Table S2. All subjects (including those belonging to the control group) signed a written informed consent and did not take any drugs at blood sampling. This study was approved by the IRCCS Regina Elena National Cancer Institute Ethical Review Board (authorization ID #CEC/541/15). Enrollment of tumor-free donors was designed in order to approach the average age and the sex distribution of the CRC cohort (described in Table S2). The age was 53.6 and the ratio M/F was 3/3.

### 4.2. Plasma Preparation

Blood (30 mL) was drawn in BD Vacutainer K_2_EDTA tubes and processed within 1h. Plasma was isolated by two successive rounds of centrifugation at 4 °C (2000× *g* for 20 min, and 16,000× *g* for 10 (min), and stored at −80 °C in single-use 2 mL aliquots until extraction [61]. These plasma preparations were expected to include exosomes, but to exclude large macrovesicles.

### 4.3. RNA Isolation

Total RNA, including miRNAs, was isolated starting from 150 µL of plasma using miRNeasy Serum/Plasma Kit (Qiagen, Hilden, Germany) according to the manufacturer’s instructions [66]. Plasma was treated to disrupt exosomes and denature miRNA-binding proteins in 5 volumes of QIAzol Lysis Reagent (Qiagen, Hilden, Germany). In total, 400 amoles of cel-miR-39-3p (Thermo Fisher Scientific, Walthman, MA, USA were added to control miRNA recovery efficiency. After the purification, total RNA was eluted in a final volume of 18 μL.

### 4.4. Next Generation Sequencing (RNA-Seq)

NGS analysis was performed at the Laboratory for Technologies of Advanced Therapies (LTTA) of Ferrara University. SmallRNA libraries were prepared from total RNA using the TruSeq^®^ Small RNA Library Prep Kit v2 (Illumina, San Diego, CA, USA, RS-200-0012/24/36/48), according to manufacturer’s indications. Briefly, 35 ng of purified RNA were linked to RNA 3′ and 5′ adapters, retrotranscribed and amplified using Illumina primers containing unique indexes for each sample. Libraries were quantified through the Agilent Bioanalyzer by using the High Sensitivity DNA kit (Agilent, Santa Clara, CA, USA, 5067-4626) and equal amounts were pooled and submitted to size-selection in order to keep only fragments between 130-160 bp. After ethanol precipitation, library pool was quantified as stated above, denatured and diluted to 1.8 pM before being sequenced using the Illumina NextSeq500 platform and NextSeq^®^ 500/550 High Output Kit v2 (75 cycles) (Illumina, San Diego, CA, USA, FC-404-2005). Raw data have been demultiplexed automatically by the Illumina BaseSpace Sequence Hub (https://basespace.illumina.com/home/index) and converted to FASTQ format. After quality checking, they were evaluated using FastQC tool (https://www.bioinformatics. babraham.ac.uk/projects/fastqc/); adapters sequences were trimmed by Cutadapt (http://cutadapt.readthedocs.io/en/stable/index.html). In this step, sequences shorter than 10 nucleotides were removed. Reads mapping was performed using the STAR algorithm (https://www.ncbi.nlm.nih.gov/pubmed/23104886), with the human microRNAs sequences from the miRBase 22 (http://www.mirbase.org/) as a reference. Raw mapped reads enumeration was performed using the htseq-count script from the HTSeq tools (http://www-huber.embl.de/HTSeq/doc/overview.html). Raw counts were normalized using DESeq2 bioconductor package (http://bioconductor.org/packages/release/bioc/html/DESeq2.html). The optimal read depth to analyze the miRNA transcriptome of plasma was determined at 10 million reads per sample [67,68]. NGS raw-data of the 9-miRNA signature are reported in Table S4.

### 4.5. ddPCR miRNA Analysis

(a) MiRNA reverse transcription: RNA extracted from plasma was reverse transcribed using the TaqMan miRNA Reverse Transcription Kit (Thermo Fisher Scientific). First, 3 µL of extracted RNA were reverse transcribed in a final volume of 20 µL, according to manufacturer’s instruction. Obtained cDNA was stored at ‒80 °C until the moment of use. (b) MiRNA RT-ddPCR: A final volume of 20 µL of ddPCR reaction was prepared according to the manufacturer’s protocol. Reaction mix was prepared, adding Supermix for Probes (no dUTP) 2 × (Bio-Rad, Hercules, CA, USA), 20 × TaqMan miRNA assay (Thermo Fisher Scientific) (assays ID are reported in Table S5) and a variable volume (depending on its levels in plasma) of cDNA. Droplets were automatically generated using Automated droplets generator (AutoDG) (Bio-Rad, Hercules, CA, USA) and the generated emulsion was amplified using the following amplification program: 95 °C for 10 min, 95 °C for 15 s and 60 °C for 1 min. Steps two and three were repeated for 40 cycles and the ramp rate was set at 2.5 °C/sec. A final step at 98 °C for 10 min to inactivate the polymerase enzyme activity was added. Amplified droplets were analyzed for the fluorescence content using QX200 Droplet Digital PCR system. Data analysis was performed using QuantaSoft software, version 1.7.4 (Bio-Rad, Hercules, CA, USA) [69,70,71]. The RT-ddPCR assay was chosen in consideration of its efficiency, the quantification of absolute amounts of analyzed molecules and the possibility to identify changes concerning low numbers of miRNA molecules, as expected using plasma preparation.

### 4.6. Statistical Analysis

In figures, the median is used to describe the miRNA expression levels, and comparisons between groups were made by using the Mann–Whitney test. Spearman’s correlation coefficient was used to detect association between miRNA plasma levels.

## 5. Conclusions

To our knowledge this is the first report, with the only noted exception of miR-483-5p [52], that provides evidence for the discrimination ability (CRC vs. tumor-free donors) of the nine miRNAs making up our signature. Overall, this signature discriminates, with a high level of sensitivity and specificity, CRC patients at an early disease stage—i.e., at surgery, representing a first step toward early molecular diagnosis. In addition, these findings contribute to an area (*KRAS*-mutated CRC) of unmet medical need under intense investigation. *KRAS*/miRNA co-targeting may alleviate the shortage of targeted therapies in this aggressive CRC subset.

In addition to the obvious impact of this study on non-invasive CRC diagnostics, our data are of interest for the development of miRNA therapeutics using anti-miRNA molecules against up-regulated miRNAs (such as miR-15b-5p, miR-584-5p and miR-425-3p) or pre-miRNA mimicking the biological functions of down-regulated miRNAs. In this respect we have recently described an antisense molecule inhibiting miR-15b-5p and inducing apoptosis on the CRC cell line HT-29 [72].

## Supplementary Materials

The following supplementary materials are available online at https://www.mdpi.com/2072-6694/12/9/2410/s1, Figure S1: Heatmap of CRC samples versus tumor-free plasmas (FC > 1.5), Figure S2: MicroRNA content in CRC plasma samples compared with the average values found in tumor-free controls, Table S1: Key examples of up-regulated miRNAs extracted from the 9-miR signature and compared with up-regulation of miR.141-3p, miR-221-3p and miR-222-3p, Table S2: Clinical data of recruited CRC patients, Table S3: Examples of molecular targets of the miRNA-signature, Table S4: NGS analysis data, Table S5: List of employed miRNA assays.
