# Supplementary material for: A Distinctive microRNA (miRNA) Signature in the Blood of Colorectal Cancer (CRC) Patients at Surgery
Source: Cancers (Basel). 2020 Aug 25;12(9):2410. doi: 10.3390/cancers12092410 (PMC7564483; doi:10.3390/cancers12092410)
Supplement: Supplementary file 1 [file cancers-12-02410-s001.pdf]

Supplementary Materials

A Distinctive microRNA (miRNA) Signature in the Blood of Colorectal Cancer (CRC) Patients at Surgery

Jessica Gasparello, Chiara Papi, Matteo Allegretti, Elena Giordani, Fabio Carboni, Settimio Zazza, Edoardo Pescarmona, Paolo Romania, Patrizio Giacomini, Chiara Scapoli, Roberto Gambari and Alessia Finotti

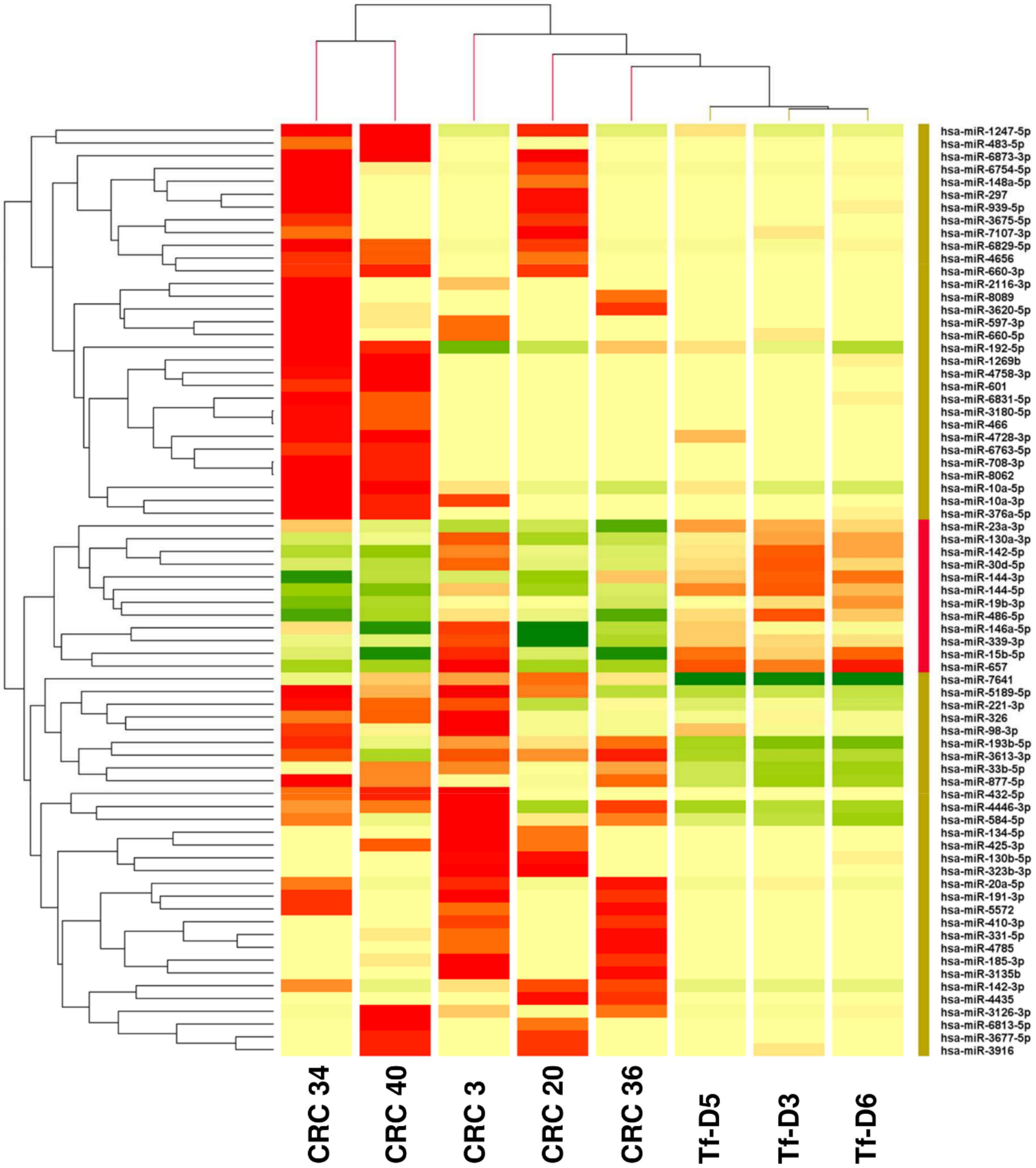

Figure S1. Heatmap of CRC samples versus tumor-free plasmas (FC > 1.5).

	miR-144-3p (copies/μL)	miR-486-5p (copies/μL)	miR-144-5p (copies/μL)	miR-15b-5p (copies/μL)	miR-1247-5p (copies/μL)	miR-584-5p (copies/μL)	miR-483-5p (copies/μL)	miR-10a-5p (copies/μL)	miR-425-3p (copies/μL)	dysregulated miRNA
**Tf-D Average ± SD**	**2.14 ± 0.85**	**494.70 ± 404.29**	**1.13 ± 0.42**	**4.63 ± 2.20**	**1.97 ± 0.81**	**0.98 ± 0.27**	**4.23 ± 1.06**	**0.54 ± 0.23**	**0.77 ± 0.58**	
**CRC 1**	**0.50**	106.40	**0.69**	**2.30**	1.40	**0.62**	3.70	**0.30** ∗	0.28	**5 (+1) ∗**
**CRC 2**	**3.10**	548.00	**3.30**	**8.50**	1.70	**2.10**	**5.90**	0.63	**1.54**	**6**
**CRC 3 #**	**1.15**	430.00	**0.55**	**42.40**	1.70	**11.00**	**5.30**	**1.30**	**22.30**	**7**
**CRC 4**	2.70	442.00	**3.00**	**33.40**	1.18	**5.70**	4.60	**1.02**	**8.20**	**5**
**CRC 5**	**0.60**	267.00	1.09	**7.20**	**0.40**	**1.80**	4.00	**0.23**	**1.80**	**6**
**CRC 6**	**8.50**	538.00	**4.70**	**11.70**	2.10	**2.00**	**7.30**	0.51	**1.90**	**6**
**CRC 7**	**0.60**	249.00	1.50	3.30	**4.10**	**2.10**	5.00	**0.77** ∗	1.10	**4 (+1) ∗**
**CRC 8**	**0.67**	239.00	1.20	5.30	2.50	**2.00**	**2.30**	0.50	0.54	**3**
**CRC 9**	**0.61**	130.00	**1.60**	**2.40**	2.30	**1.70**	**2.90**	**0.16**	0.68	**6**
**CRC 10**	**0.45**	108.70	**3.00**	2.80	**0.98**	**1.60**	**2.00**	**0.08**	0.33	**6**
**CRC 11**	**0.86**	143.90	0.95	3.50	**1.14**	**1.32**	4.20	0.51	0.72	**3**
**CRC 12**	**3.8**	745.00	**6.40**	**9.70**	1.60	**2.00**	**5.90**	0.44	0.59	**5**
**CRC 13**	**0.07**	**60.00**	**0.70**	5.80	**3.30**	**1.70**	4.10	**0.22**	1.15	**6**
**CRC 14**	**0.76**	90.70	**0.46**	**2.10**	**0.85**	**1.41**	**5.30**	**0.14**	0.19	**7**
**CRC 15**	**0.54**	137.80	0.82	**2.10**	1.30	**1.60**	**6.30**	0.37	0.51	**4**
**CRC 16**	**0.42**	176.00	**0.42**	3.30	1.80	1.25	**3.10**	0.52	0.19	**3**
**CRC 17**	**0.43**	146.00	0.72	4.70	1.70	**2.00**	**2.80**	0.47	0.87	**3**
**CRC 18**	**4.40**	**955.00**	**2.10**	**7.00**	**0.58**	**2.20**	3.20	0.35	0.95	**6**
**CRC 19**	**0.21**	173.00	**0.43**	4.50	**3.80**	**1.60**	**2.90**	0.66	0.91	**5**
**CRC 20 #**	**3.40**	330.00	**3.30**	**11.10**	1.80	**1.90**	4.80	**1.09**	**2.70**	**6**
**CRC 21**	**1.21**	307.00	0.92	**7.80**	1.70	**1.90**	4.10	0.57	**1.90**	**4**
**CRC 22**	**1.27**	177.00	**2.90**	**10.60**	2.40	**2.90**	**7.00**	**0.13**	**1.50**	**7**
**CRC 23**	**0.82**	158.00	1.01	6.50	**0.81**	**0.63**	**1.70**	0.38	**1.39**	**5**
**CRC 24**	**1.00**	186.00	**2.6**	**7.00**	1.50	**0.58**	**5.30**	0.47	0.48	**5**
**CRC 26**	**0.41**	152.00	**0.63**	**2.40**	**0.99**	**0.48**	4.10	0.54	0.38	**5**
**CRC 27**	2.40	**1978.00**	**2.70**	**10.20**	1.70	**2.30**	**2.10**	**0.26**	**1.90**	**7**
**CRC 28**	1.90	456.00	**2.60**	5.60	**0.68**	**0.46**	**2.00**	0.38	0.56	**4**
**CRC 29**	1.60	402.00	0.84	4.40	**0.62**	0.91	3.40	**0.13**	0.61	**2**
**CRC 30**	1.70	271.00	0.96	3.00	1.26	0.94	3.60	0.38	0.47	**0**
**CRC 31**	**0.52**	272.00	0.90	**1.50**	**1.15**	0.78	**2.30**	0.36	0.53	**4**
**CRC 33**	**0.24**	**87.50**	0.82	5.40	**2.80**	**1.70**	4.80	0.70	**1.90**	**5**
**CRC 34 #**	**1.00**	201.00	**0.45**	5.80	**10.00**	**6.10**	**8.80**	**1.90**	**2.70**	**7**
**CRC 36 #**	**1.10**	**71.80**	**0.24**	**8.60**	**1.10**	1.20	**2.00**	**0.27** ∗	**1.55**	**8 (+1) ∗**
**CRC 40 #**	**0.81**	**76.70**	**0.50**	2.80	**7.20**	**2.50**	**36.60**	0.33	0.79	**6**
**CRC 46**	2.10	376.00	0.89	4.70	**4.70**	**1.80**	3.40	**0.82**	**1.70**	**4**
**CRC Average ± SD**	**1.48 ± 1.63**	**319.64 ± 350.31**	**1.60 ± 1.39**	**7.41 ± 8.20**	**2.14 ± 1.93**	**2.08 ± 1.95**	**5.05 ± 5.75**	**0.51 ± 0.37**	**1.88 ± 3.81**	

Figure S2. MicroRNA content in CRC plasma samples compared with the average values found in tumor-free controls. Plasma miRNAs levels (copies/μL) in CRC (from CRC1 to CRC46) plasma samples, detected by dd-RT-PCR. Dys-regulation of the miRNAs was boxed when an increase/decrease equal or higher of the Tf-D average ± SD was detected. In green boxes the down-regulated miRNA values (values < average of Tf-D ± SD); in red boxes the up-regulated miRNA values (values > average of Tf-D ± SD). Asterisk indicates newly detected samples with the respect to Table 1 proposed in main text. The number of dys-regulated miRNAs is indicated for each CRC sample in the last right column. CRC and Tf-D plasma samples analyzed both by NGS and dd-RT-PCR were marked with hashtag (#).

Table S1. Key examples of up-regulated miRNAs extracted from the 9-miR signature and compared with up-regulation of miR-141-3p, miR-221-3p and miR-222-3p.

	**miR-15b-5p (copies/µL)**	**miR-584-5p (copies/µL)**	**miR-425-3p (copies/µL)**	**dysregulated miRNA**	**miR-141-3p (copies/µL)**	**miR-221-3p (copies/µL)**	**miR-222-3p (copies/µL)**	**dysregulated miRNA**	**total dysregulated miRNA**
**Tf-D Average ± SD**	**4.63 ± 2.20**	**0.98 ± 0.27**	**0.77 ± 0.58**		**9.3 ± 0.71**	**8.1 ± 1.85**	**60.2 ± 4.86**		
**CRC 1**	2.30	0.62	0.28	**0**	5.90	**10.30**	**66.10**	**2**	**2**
**CRC 2**	**8.50**	**2.10**	**1.54**	**3**	6.60	8.90	63.10	**0**	**3**
**CRC 3 #**	**42.40**	**11.00**	**22.30**	**3**	8.80	**122.00**	**106.00**	**2**	**5**
**CRC 4**	**33.40**	**5.70**	**8.20**	**3**	8.20	**52.00**	**95.00**	**2**	**5**
**CRC 5**	**7.20**	**1.80**	**1.80**	**3**	7.70	**12.20**	63.10	**1**	**4**
**CRC 6**	**11.70**	**2.00**	**1.90**	**3**	7.10	9.20	64.30	**0**	**3**
**CRC 7**	3.30	**2.10**	1.10	**1**	7.40	6.50	64.70	**0**	**1**
**CRC 8**	5.30	**2.00**	0.54	**1**	5.80	**10.70**	**65.10**	**2**	**3**
**CRC 9**	2.40	**1.70**	0.68	**1**	7.10	5.20	61.50	**0**	**1**
**CRC 10**	2.80	**1.60**	0.33	**1**	**18.40**	6.90	60.20	**1**	**2**
**CRC 11**	3.50	**1.32**	0.72	**1**	7.90	**13.20**	56.50	**1**	**2**
**CRC 12**	**9.70**	**2.00**	0.59	**2**	7.30	9.30	59.00	**0**	**2**
**CRC 13**	5.80	**1.70**	1.15	**1**	9.60	**12.20**	**66.80**	**2**	**3**
**CRC 14**	2.10	**1.41**	0.19	**1**	7.40	5.80	60.90	**0**	**1**
**CRC 15**	2.10	**1.60**	0.51	**1**	7.30	8.20	64.30	**0**	**1**
**CRC 16**	3.30	1.25	0.19	**0**	9.80	6.10	52.10	**0**	**0**
**CRC 17**	4.70	**2.00**	0.87	**1**	**14.30**	8.60	59.30	**1**	**2**
**CRC 18**	**7.00**	**2.20**	0.95	**2**	9.70	9.00	62.50	**0**	**2**
**CRC 19**	4.50	**1.60**	0.91	**1**	9.30	**19.80**	**67.80**	**2**	**3**
**CRC 20 #**	**11.10**	**1.90**	**2.70**	**3**	9.40	**12.50**	61.50	**1**	**4**
**CRC 21**	**7.80**	**1.90**	**1.90**	**3**	7.40	**11.00**	47.80	**1**	**4**
**CRC 22**	**10.60**	**2.90**	**1.50**	**3**	9.90	**13.20**	63.20	**1**	**4**
**CRC 23**	6.50	0.63	**1.39**	**1**	**10.10**	**12.80**	57.20	**2**	**3**
**CRC 24**	**7.00**	0.58	0.48	**1**	**12.30**	6.80	22.30	**1**	**2**
**CRC 26**	2.40	0.48	0.38	**0**	**12.20**	5.00	58.70	**1**	**1**
**CRC 27**	**10.20**	**2.30**	**1.90**	**3**	**10.30**	**10.10**	**71.60**	**3**	**6**
**CRC 28**	5.60	0.46	0.56	**0**	9.70	7.20	43.20	**0**	**0**
**CRC 29**	4.40	0.91	0.61	**0**	**11.70**	**11.80**	31.40	**2**	**2**
**CRC 30**	3.00	0.94	0.47	**0**	7.80	6.40	**66.20**	**1**	**1**
**CRC 31**	1.50	0.78	0.53	**0**	9.00	8.70	**65.40**	**1**	**1**
**CRC 33**	5.40	**1.70**	**1.90**	**2**	**11.70**	**18.60**	55.60	**2**	**4**
**CRC 34 #**	5.80	**6.10**	**2.70**	**2**	**20.00**	4.30	**321.00**	**2**	**4**
**CRC 36 #**	**8.60**	1.20	**1.55**	**2**	**12.40**	6.10	**277.00**	**2**	**4**
**CRC 40 #**	2.80	**2.50**	0.79	**1**	**19.90**	1.90	**270.00**	**2**	**3**
**CRC 46**	4.70	**1.80**	**1.70**	**2**	**18.10**	3.40	**323.00**	**2**	**4**
**CRC Average ± SD**	**7.41 ± 8.20**	**2.08 ± 1.95**	**1.88 ± 3.81**		**10.21 ± 3.80**	**13.60 ± 20.56**	**88.38 ± 78.00**		

Table S2. Clinical data of recruited CRC patients. NA = not available; ADC = Adenocarcinoma; # mutation detected in plasma samples; * mutations detected in tumor tissue. Tumor localization, tumor size and TNM classification are reported in the table: pT = size of the primary tumor and grade of nearby tissue invasion; pN = grade of regional lymph nodes involvement; M = presence of distant metastasis.

Patient ID	Sex	Age (years)	Tumor localization	Diagnosis	pT	pN	M	Grading	Tumor volume (diameter)	KRAS#	KRAS*	NRAS*	BRAF*
**CRC 1**	M	63	Colon	Mucinous ADC of colon	3	2	1	NA	3.5 cm	WT	WT	WT	WT
**CRC 2**	M	54	Rectum	ADC of rectum	0	0	0	NA	NA	NA	WT	WT	WT
**CRC 3**	F	65	Colon	Mucinous ADC of colon	2	0	0	1	1.8 cm	G12D	G12D	WT	WT
**CRC 4**	F	72	Colon	ADC of colon	1	0	0	1	4.7 cm	G12D	G12D	WT	WT
**CRC 5**	M	36	Colon	ADC of colon	4a	1b	1	3	5.0 cm	G60D	WT	WT	WT
**CRC 6**	M	75	Colon	ADC of right colon	4a	2b	1	3	2.0 cm	WT	WT	WT	V600E
**CRC 7**	M	51	Sigmoid colon	ADC of sigmoid colon	3	0	0	2	4.5 cm	WT	WT	WT	WT
**CRC 8**	M	42	Sigmoid colon	ADC of sigmoid colon	2	0	0	2	3.0 cm	WT	WT	WT	WT
**CRC 9**	M	60	Sigmoid colon	ADC of sigmoid colon	4a	0	0	2	3.0 cm	WT	WT	WT	WT
**CRC 10**	F	78	Rectum	ADC of rectum	3	0	0	2	3.0 cm	WT	WT	WT	WT
**CRC 11**	M	76	Sigmoid colon	ADC of sigmoid colon	3	0	0	3	5.0 cm	G12A	G12A	WT	WT
**CRC 12**	M	50	Colon	ADC of right colon	3	1a	1	2	4.5 cm	WT	WT	WT	WT
**CRC 13**	F	83	Colon	ADC of right colon	3	0	0	2	4.0 cm	WT	WT	WT	V600E
**CRC 14**	F	76	Rectum	ADC of rectum	2	1b	0	3	3.0 cm	WT	WT	WT	WT
**CRC 15**	F	65	Colon	ADC of colon	2	0	0	2	6.0 cm	NA	WT	WT	WT
**CRC 16**	M	77	Colon	ADC of left colon	3	0	0	2	3.5 cm	NA	WT	WT	WT
**CRC 17**	F	54	Colon	ADC of colon	4a	1b	0	2	5.0 cm	G12D	G12D	WT	WT
**CRC 18**	M	75	Colon	ADC of colon	3	1b	0	2	4.0 cm	A146T	A146T	WT	WT
**CRC 19**	M	82	Colon	ADC of right colon	2	0	0	2	3.5 cm	WT	WT	WT	WT
**CRC 20**	M	72	Colon	ADC of right colon	3	0	0	2	4.0 cm	NA	WT	WT	WT
**CRC 21**	M	78	Colon	ADC of right colon	3	0	0	2	4.5 cm	G13D	G13D	WT	WT
**CRC 22**	M	77	Colon	ADC of right colon	3	0	0	2	4.5 cm	G12V	G12V	WT	WT
**CRC 23**	M	64	Rectum	ADC of rectum	4a	0	0	2	3.0 cm	G12V	G12V	WT	WT
**CRC 24**	M	52	Sigmoid colon	Mucinous ADC of sigmoid colon	4b	1a	0	3	4.0 cm	G12D	G12D	WT	WT
**CRC 26**	M	83	Rectum	ADC of rectum	2	1b	0	2	4.0 cm	NA	WT	WT	WT
**CRC 27**	F	85	Colon	Villous tubular adenoma	1	0	0	1	2.5 cm	G12R	G12R	WT	WT
**CRC 28**	M	53	Colon	ADC of right colon	3	0	0	2	5.0 cm	G13D	G13D	WT	WT
**CRC 29**	M	47	Rectum	ADC of rectum	1	0	0	2	< 1.0 mm	WT	WT	WT	WT
**CRC 30**	M	71	Rectum	ADC of right colon	3	0	0	2	2.5 cm	NA	WT	WT	WT
**CRC 31**	M	83	Colon	ADC of right colon	3	1	0	2	4.0 cm	G12D	G12D	WT	WT
**CRC 33**	M	55	Peritoneal carcinosis	Mixed adeno-neuroendocrine carcinoma (MANEC)	4	0	0	mixed	5.0 cm	G12V	G12V	WT	WT
**CRC 34**	F	74	Colon	Metastatic ADC of colon	4	1	0	3	NA	G13D	G13D	WT	WT
**CRC 36**	F	44	Rectum	ADC of rectum	3	1	0	3	NA	NA	G13D	WT	WT
**CRC 40**	F	79	Colon	Metastatic ADC of colon	4	2	1	3	NA	G12A	G12A	WT	WT
**CRC 46**	F	59	Colon	Metastatic ADC of colon	4	1	NA	2	NA	NA	WT	WT	WT

Table S3. Examples of molecular targets of the miRNA-signature.

MicroRNA	Validated molecular target	Reference
miR-144-3p	***mTOR**:* miR-144-3p suppresses proliferation and induces apoptosis of osteosarcoma cellular models.<br><br>***PRR11**:* miR-144-3p induces cell cycle arrest and apoptosis in pancreatic cancer cells.<br><br>***BCL6**:* miR-144-3p inhibits cell proliferation of colorectal cancer cells.	*Ren et al., 2018* [1]<br><br>*Li et al., 2017* [2]<br><br>*Sun et al., 2020* [3]
miR-486-5p	***PIK3R1**:* miR-486-5p regulates the migration and invasion of colorectal cancer cellular models.	*Zhang et al., 2018* [4]
miR-144-5p	**CCNE1 and CCNE2:** miR-144-5p regulates cell cycle in bladder cancer models.	*Matsushita et al., 2015* [5]
miR-15b-5p	***HPSE2**:* miR-15b-5p promotes growth and metastasis in breast cancer models.<br><br>***Axin2:*** miR-15b-5p promote cell proliferation and invasion in liver cancer<br><br>***RECK:*** miR-15b-5p facilitates the tumorigenicity predicts tumour recurrence in prostate cancer	*Wu et al., 2020* [6]<br><br>*Dong et al., 2019* [7]<br><br>*Chen et al., 2018* [8]
miR-1247-5p	**Wnt3:** miR-1247-5p functions as a tumor suppressor in human hepatocellular carcinoma models.	*Chu et al., 2017* [9]
miR-584-5p	***ROCK-1:*** miR-584-5p regulates invasion and migration thyroid carcinoma models.	*Xiang et al., 2015* [10]
miR-483-5p	***ERK1:*** miR-483–5p suppresses the proliferation and induced a G0/G1 arrest of glioma cells.	Wang et al., 2012 [11]
miR-10a-5p	***MMP14:*** miR-10a-5p suppresses colorectal cancer metastasis regulating the epithelial-to-mesenchymal transition.	Liu et al., 2017 [12]
miR-425-3p	**AKT1*:*** miR-425-3p confers cisplatin resistance in NSCLC.	Ma et al., 2019 [13]

Table S4. NGS analysis data.

**miRNAs differentially expressed in CRC Vs Tf-D**				
miRNAs expression is normalized and log2 transformed				
# Notes: Created from Advanced Analysis operation: significance Analysis.				
#Experiment: CRC-project_smallRNAseq				
#corrected p-value cut-off:0.45				
#Fold change cut-off:2.0				
#Selected Test:	*Moderated T-Test*			
#Entity List:	*Fold change >= 2.0 CRC Vs Tf-D*			
#Interpretation:	*Type*			
#p-value computation:	*Asymptotic*			
#Multiple Testing Correction:	***Benjamini-Hochberg***			
**miRNA ID**	**p (Corr) ([CRC] Vs [Tf-D])**	**p ([CRC] Vs [Tf-D])**	**Regulation ([CRC] Vs [Tf-D])**	**FC (abs) ([CRC] Vs [Tf-D])**
hsa-miR-10a-5p	0,4018353	0,24126972	up	2,8678014
hsa-miR-1247-5p	0,4018353	0,19052348	up	7,9928136
hsa-miR-144-3p	0,3119489	0,01949680	down	2,7872958
hsa-miR-144-5p	0,1726657	0,00863329	down	2,6813111
hsa-miR-15b-5p	0,4018353	0,16873904	down	2,4655366
hsa-miR-425-3p	0,4018353	0,21251409	up	2,1048949
hsa-miR-483-5p	0,4355741	0,38219790	up	2,5161827
hsa-miR-486-5p	0,3196733	0,02797141	down	2,5935428
hsa-miR-584-5p	0,4018353	0,06719047	up	2,9576976

Table 5. List of employed miRNA assays.

miRNA name	Assay ID
hsa-miR-10a-5p	000387
hsa-miR-1247-5p	002898
hsa-miR-141-3p	000463
hsa-miR-144-3p	002676
hsa-miR-144-5p	002148
hsa-miR-15b-5p	000390
hsa-miR-221-3p	000524
hsa-miR-222-3p	002676
hsa-miR-425-3p	002302
hsa-miR-483-5p	002328
hsa-miR-486-5p	001278
hsa-miR-584-5p	001624
cel-miR-39-3p	000200

Reference

1. Ren, Y.F.; Zhang, T.H.; Zhong, S.; Zhao, Y.T.; Lv, Y.N. miR-144 suppresses proliferation and induces apoptosis of osteosarcoma cells via direct regulation of mTOR expression. *Oncol Lett* **2018**, *15*, 1163–1169.
2. Li, J.; Sun, P.; Yue, Z.; Zhang, D.; You, K.; Wang, J. miR-144-3p Induces Cell Cycle Arrest and Apoptosis in Pancreatic Cancer Cells by Targeting Proline-Rich Protein 11 Expression via the Mitogen-Activated Protein Kinase Signaling Pathway. *DNA Cell Biol* **2017**, *36*, 619–626.
3. Sun, N.; Zhang, L.; Zhang, C.; Yuan, Y. miR-144-3p inhibits cell proliferation of colorectal cancer cells by targeting BCL6 via inhibition of Wnt/β-catenin signalling. *Cell Mol Biol Lett* **2020**, *25*, 19.
4. Zhang, Y.; Fu, J.; Zhang, Z.; Qin, H. miR-486-5p regulates the migration and invasion of colorectal cancer cells through targeting PIK3R1. *Oncol Lett* **2018**, *15*, 7243–7248.
5. Matsushita, R.; Seki, N.; Chiyomaru, T.; Inoguchi, S.; Ishihara, T.; Goto, Y.; Nishikawa, R.; Mataki, H.; Tatarano, S.; Itesako, T.; Nakagawa, M.; Enokida, H. Tumour-suppressive microRNA-144-5p directly targets CCNE1/2 as potential prognostic markers in bladder cancer. *Br J Cancer* **2015**, *113*, 282–289.
6. Wu, B.; Liu, G.; Jin, Y.; Yang, T.; Zhang, D.; Ding, L.; Zhou, F.; Pan, Y.; Wei, Y. miR-15b-5p Promotes Growth and Metastasis in Breast Cancer by Targeting HPSE2. *Front Oncol* **2020**, *10*, 108.
7. Dong, Y.; Zhang,N.; Zhao, S.; Chen, X.; Li, F.; Tao, X. miR-221-3p and miR-15b-5p promote cell proliferation and invasion by targeting Axin2 in liver cancer. *Oncol Lett* **2019**, *18*, 6491–6500.
8. Chen, R.; Sheng, L.; Zhang, H.J.; Ji, M.; Qian, W.Q. miR-15b-5p facilitates the tumorigenicity by targeting RECK and predicts tumour recurrence in prostate cancer. *J Cell Mol Med* **2018**, *22*, 1855–1863.
9. Chu, Y.; Fan, W.; Guo, W.; Zhang, Y.; Wang, L.; Guo, L.; Duan, X.; Wei, J.; Xu, G. miR-1247-5p functions as a tumor suppressor in human hepatocellular carcinoma by targeting Wnt3. *Oncol Rep* **2017**, *38*, 343–351.
10. Xiang, J.; Wu, Y.; Li, D.S.; Wang, Z.Y.; Shen, Q.; Sun, T.Q.; Guan, Q.; Wang, J.Y. miR-584 Suppresses Invasion and Cell Migration of Thyroid Carcinoma by Regulating the Target Oncogene ROCK1. *Oncol Res Treat* **2015**, *38*, 436–440.
11. Wang, L.; Shi, M.; Hou, S.; Ding, B.; Liu, L.:, Ji, X.; Zhang, J.; Deng, Y. MiR-483-5p suppresses the proliferation of glioma cells via directly targeting ERK1. FEBS Lett **2012**, *586*, 1312–1317.
12. Liu, Y.; Zhang, Y.; Wu, H.; Li, H.; Zhang, Y.; Liu, M.; Li, X.; Tang, H. miR-10a suppresses colorectal cancer metastasis by modulating the epithelial-to-mesenchymal transition and anoikis. Cell Death Dis **2017**, *8*, e2739.
13. Ma, Y.; Yuwen, D.; Chen, J.; Zheng, B.; Gao, J.; Fan, M.; Xue, W.; Wang, Y.; Li, W.; Shu, Y.; Xu, Q.; Shen, Y. Exosomal Transfer Of Cisplatin-Induced miR-425-3p Confers Cisplatin Resistance In NSCLC Through Activating Autophagy. *Int J Nanomedicine* **2019**, *14*, 8121–8132.

© 2020 by the authors. Licensee MDPI, Basel, Switzerland. This article is an open access article distributed under the terms and conditions of the Creative Commons Attribution (CC BY) license (http://creativecommons.org/licenses/by/4.0/).
